# Negative Auto-Regulation of Myostatin Expression is Mediated by Smad3 and MicroRNA-27

**DOI:** 10.1371/journal.pone.0087687

**Published:** 2014-01-31

**Authors:** Craig McFarlane, Anuradha Vajjala, Harikumar Arigela, Sudarsanareddy Lokireddy, XiaoJia Ge, Sabeera Bonala, Ravikumar Manickam, Ravi Kambadur, Mridula Sharma

**Affiliations:** 1 Singapore Institute for Clinical Sciences, Agency for Science, Technology and Research, Singapore, Singapore; 2 School of Biological Sciences, Nanyang Technological University, Singapore, Singapore; 3 Department of Biochemistry, Yong Loo Lin School of Medicine, National University of Singapore, Singapore, Singapore; Johns Hopkins University School of Medicine, United States of America

## Abstract

Growth factors, such as myostatin (Mstn), play an important role in regulating post-natal myogenesis. In fact, loss of Mstn has been shown to result in increased post-natal muscle growth through enhanced satellite cell functionality; while elevated levels of Mstn result in dramatic skeletal muscle wasting through a mechanism involving reduced protein synthesis and increased ubiquitin-mediated protein degradation. Here we show that miR-27a/b plays an important role in feed back auto-regulation of Mstn and thus regulation of post-natal myogenesis. Sequence analysis of *Mstn* 3′ UTR showed a single highly conserved miR-27a/b binding site and increased expression of miR-27a/b was correlated with decreased expression of *Mstn* and vice versa both *in vitro* and in mice *in vivo*. Moreover, we also show that *Mstn* gene expression was regulated by miR-27a/b. Treatment with miR-27a/b-specific AntagomiRs resulted in increased *Mstn* expression, reduced myoblast proliferation, impaired satellite cell activation and induction of skeletal muscle atrophy that was rescued upon either blockade of, or complete absence of, Mstn. Consistent with this, miR-27a over expression resulted in reduced *Mstn* expression, skeletal muscle hypertrophy and an increase in the number of activated satellite cells, all features consistent with impaired Mstn function. Loss of *Smad3* was associated with increased levels of Mstn, concomitant with decreased miR-27a/b expression, which is consistent with impaired satellite cell function and muscular atrophy previously reported in *Smad3*-null mice. Interestingly, treatment with Mstn resulted in increased miR-27a/b expression, which was shown to be dependent on the activity of Smad3. These data highlight a novel auto-regulatory mechanism in which Mstn, via Smad3 signaling, regulates miR-27a/b and in turn its own expression. In support, Mstn-mediated inhibition of *Mstn* 3′ UTR reporter activity was reversed upon miR-27a/b-specific AntagomiR transfection. Therefore, miR-27a/b, through negatively regulating *Mstn*, plays a role in promoting satellite cell activation, myoblast proliferation and preventing muscle wasting.

## Introduction

Myostatin (Mstn) is a secreted growth factor that belongs to the TGF-β super-family. Mstn is produced predominantly in skeletal muscle with lower levels of expression observed in white adipose tissue [1], heart [2] and mammary gland [3]. Analysis has revealed that Mstn is a profound negative regulator of muscle growth; while inactivation or mutation of the *Mstn* gene leads to increased skeletal muscle mass [1], [4], enhanced myoblast proliferation [5], differentiation [6] and improved skeletal muscle regeneration [7], increased levels of Mstn result in severe cachectic-like muscle wasting [8]–[11].

The expression of *Mstn* is initially detected at embryonic day 9.5 in developing somites and continues to be detected postnataly in adult skeletal muscle fibers [1]. Furthermore, the mRNA expression of *Mstn* is developmentally regulated. While relatively abundant expression of *Mstn* is observed during fetal development, following birth the expression of *Mstn* rapidly decreases and remains quite low during postnatal development [12]. Previous work has also demonstrated that in adult skeletal muscle the expression of Mstn is greater in fast-twitch when compared to slow-twitch muscles [13], [14] and thus is speculated to play a role in regulating muscle fiber type. Although we know that the expression of Mstn is regulated during myogenesis the exact mechanisms through which the abundance of Mstn is regulated remain to be fully determined. However, work from our lab has revealed that *Mstn* is transcriptionally regulated by the transcription factor MyoD through E-Box elements contained within the enhancer region of the *Mstn* gene [15]. In addition, further work has demonstrated that Mstn is able to negatively auto-regulate its own expression through a Smad7-dependent mechanism [16]. In eukaryotes, transcriptional regulation is not the only mechanism through which gene expression is controlled. Most notably, post transcriptional regulation of mRNA stability and translation through the action of microRNAs (miRNAs) plays a major role in regulating mRNA abundance. miRNAs are short single stranded RNAs that can bind specifically to complementary sequences found in 3′ untranslated regions (UTR) of target mRNAs, resulting in either repression of translation or degradation of mRNAs through the RNA-induced silencing complex (RISC) [17], [18]. Previously published work has revealed that Mstn levels are regulated by miRNAs. Specifically, transgenic over expression of miR-208a in the heart has been shown to result in cardiac hypertrophy together with reduced expression of *Mstn* [19]. Furthermore, over expression of miR-499 leads to reduced *Mstn* 3′UTR activity, suggesting that *Mstn* is a target of miR-499 [20]. In addition, essential amino acids have been shown to promote muscle hypertrophy by not only suppressing Mstn levels but by inducing greater expression of miR-499, -208b, -23a, -1, and -206 [21]. Interestingly, a naturally occurring gain of function mutation in the 3′UTR region of the Texel sheep *Mstn* gene creates a miR-206 site causing translational inhibition of Mstn expression and a double muscling phenotype [22]. More recently, microRNA-27 (miR-27) has been shown to target and inhibit Mstn. Work by Allen and Loh revealed that miR-27a/b is able to reduce *Mstn* expression and mRNA stability and moreover indicated miR-27a/b may play a role in the increased expression of *Mstn* observed firstly, in Fast-twitch muscle, when compared to slow-twitch muscle and secondly, in response to Dexamethasone treatment [23]. Similarly, Huang *et al* revealed that over expression of miR-27a through addition of miR-27a mimics resulted in reduced *Mstn* mRNA expression and increased myoblast proliferation [24], consistent with known Mstn function.

Here we now show further evidence to support that *Mstn* gene expression is regulated by the miR-27a/b, as such AntagomiRs against miR-27a/b were able to increase *Mstn* expression, reduce myoblast proliferation and induce myotubular atrophy. Importantly, AntagomiR-27a/b-mediated myotube atrophy was due to increased Mstn function, as either blockade or complete absence of *Mstn* rescued the myotubular atrophy. Furthermore, results confirm a role for miR-27a/b in regulating muscle fiber type-specific and tissue-specific expression of *Mstn* and suggest that miR-27a/b may play a role in regulating *Mstn* expression and thus function during myogenic differentiation. We further show the utility of miR-27a/b in regulating *Mstn* expression and activity *in vivo* and that in *Smad3*-null mice there is increased expression of *Mstn*, which is due to reduced endogenous miR-27a/b expression in these mice. Results also reveal for the first time that Mstn up regulates the expression of miR-27a/b via Smad3, which in turn targets and represses *Mstn*, forming the basis of a novel microRNA-mediated Mstn negative auto-regulatory loop during myogenesis.

## Materials and Methods

### Ethics Statement

All experiments involving animals were approved by the Nanyang Technological University Institutional Animal Care and Use Committee (NTU-IACUC), Singapore (Approval Number: ARF SBS/NIE-A 0057). All surgery was performed under Ketamine/Xylazine anesthesia and all efforts were made to minimize animal suffering.

### Animals

Four-six-week-old C57BL/6J male wild type (WT) mice were obtained from the National University of Singapore Centre for Animal Resources, Singapore. *Myostatin*-null mice (*Mstn* ^−/+^) were gifted by Prof. See-Jin Lee (Johns Hopkins University, Baltimore, MD, USA). All mice were housed in groups at a constant temperature (20°C) under a 12 h/12 h artificial light/dark cycle with free access to water. To study the effect of miR-27a blockade *in vivo*, 20 nM (25 µl total volume; in sterile nuclease free water) of each AntagomiR (AntagomiR-27a or AntagomiR Neg) oligonucleotides [25], was injected into the *M. tibialis anterior* (TA) muscle of anaesthetized WT mice (n = 3) using a 28-gauge syringe (Hamilton Co., Reno, NV, USA). The contralateral limb of each mouse was injected with negative control AntagomiR (AntagomiR Neg). *In vivo* transfection of plasmid DNA (pcDNA-miR-27a or pcDNA-miR-neg) was performed by intramuscular injection of 25 µg (25 µl total volume; in sterile PBS) of each plasmid DNA into the TA muscle of anaesthetized mice. The contralateral limb of each mouse was injected with the empty vector (pcDNA-miR-neg) as a control. Electrical pulses (50 Volts/cm, 5 pulses, 200 ms intervals) were then applied with two platinum electrodes placed on either side of the muscle belly, using the ECM 830 Electroporation system (BTX Instrument Division, Harvard Apparatus, Inc. MA, USA). Eight days post-injection, *M. tibialis anterior* (TA) muscles from both sides of the hind-limb were harvested for histological and molecular analysis. For staining of skeletal muscle sections, TA muscles were covered with OCT compound and then frozen in isopentane cooled with liquid nitrogen. Transverse sections (8 µm) were cut from the mid-belly of the muscle and mounted on slides for hematoxylin and 1% eosin (H&E) staining and for detection of MyoD and Pax7 by immunocytochemistry. Images were captured using the Leica CTR 6500 microscope equipped with the Leica DFC 420 camera and Image Pro Plus software (Media Cybernetics, Bethesda, MD). Muscle fiber size was measured as Cross Sectional Area (CSA) from 500 myofibers per mouse (n = 3).

### C2C12 myoblast cell culture

Mouse C2C12 myoblasts, obtained from American Type Culture Collection (Manassas, VA, USA), were maintained as previously described [26]. Assessment of C2C12 myoblast proliferation was performed as previously described [6], [26]. Briefly, C2C12 myoblasts were seeded at a density of 1000 cells/well in 96-well plates in proliferation medium (DMEM, 10% FBS and 1% P/S; Invitrogen, Carlsbad, CA, USA). After an overnight attachment period, myoblasts were transfected with 25 nM each of AntagomiRs specific for miR-27a (AntagomiR-27a), miR-27b (AntagomiR-27b) or negative control AntagomiR (AntagomiR Neg) (Dharmacon Inc, USA) using Lipofectamine 2000 (LF2000; Invitrogen, USA), as per the manufacturer's guidelines. Following a further period of 72 h growth, proliferation was assessed using the methylene blue photometric end-point assay, as previously described [27], where absorbance at 655 nm is directly proportional to final cell number. To assess the effect of miR-27a/b blockade on differentiated myotubes and to study the effect of Mstn blockade on AntagomiR-27a/b-mediated myotube atrophy, C2C12 myoblasts were induced to differentiate on Thermanox coverslips under low-serum conditions (DMEM, 2% Horse Serum) for 24 h. Following this, 24 h differentiated C2C12 myoblasts were transfected, using LF2000, with 50 nM each of AntagomiR-27a, AntagomiR-27b or AntagomiR Neg. Twelve hours following transfection, cells were then treated with either vehicle (Dialysis buffer; DB) or with a soluble form of the Activin receptor Type IIB (sActRIIB) at a final concentration of 3 µg/ml and allowed to differentiate for 72 h. The expression and purification of the sActRIIB Mstn antagonist was performed as previously described [28]. Differentiated Myotubes were then fixed with ethanol∶formaldehyde∶glacial acetic acid (20∶2∶1) and stained with H&E. Images of the cultures were then captured and myotube area assessed. Mstn-overexpressing CHO cells were kindly gifted by Dr. Se-Jin Lee, Johns Hopkins University, USA. Mstn-overexpressing CHO cells were propagated and Mstn protein containing conditioned medium (CMM) was collected as described previously [11]. The final concentration of Mstn protein present in all CMM treatments was 10 ng/ml, as estimated by ELISA (Immundiagnostik, Bensheim, Germany). Conditioned medium collected from control CHO cells (CCM) was used as a control for CMM treatment experiments. For Mstn treatment, C2C12 myoblasts were either grown for 16 h or were differentiated for 48 h before further treatment with either CMM or CCM for 12 h.

### Primary myoblast culture

Mouse primary myoblasts were cultured from hind-limb muscles isolated from WT and Mstn^−/−^ mice using a modified method of Partridge TA [29]. Briefly, hind-limb muscles were excised, minced and then digested in 0.2% collagenase type 1A for 90 min. Fibroblasts were removed by pre-plating the cells on uncoated plates for 3 h at 37°C 5% CO_2_. Primary myoblasts were cultured on 10% Matrigel (BD Biosciences) coated plates and were maintained in proliferation medium, (DMEM, 20% FBS, 10% HS, 1% P/S and 1% Chicken Embryo Extract) at 37°C 5% CO_2_. Primary myoblasts were induced to differentiate, transfected with either AntagomiR-27a or AntagomiR Neg and fixed and stained with H&E, as described above. Images of the cultures were then captured and myotube area assessed.

### Specific Inhibitor of SMAD3 (SIS3) treatment

C2C12 myoblasts were differentiated (as described above) for 48 h followed by a further 24 h in the absence (0.05% DMSO) or presence of the SMAD3-specific inhibitor SIS3 (10 µM; Sigma-Aldrich). Cells were then harvested for total RNA isolation and subsequent quantitative real-time PCR (qPCR) analysis.

### Detection of MyoD and Pax7 by immunofluorescence

Muscle sections were fixed in 4% paraformaldehyde for 5 min and then permeabilized in 0.2% PBS-Tween 20. After this, sections were blocked in a solution containing 6% mouse IgG blocking reagent (MOM Immunodetection kit; Vector laboratories, Inc, CA, USA) and 3% bovine serum albumin (BSA) in PBS for 1 h, followed by 5 min in MOM protein diluent with 1.5% BSA in PBS, as per the manufacturer's instruction. Muscle sections were then stained with mouse monoclonal anti-Pax7 (Developmental Studies Hybridoma Bank; DSHB, Iowa City, IA, USA; 1∶1000) primary antibody in 1.5% BSA in PBS overnight at 4°C. Following incubation with horse biotinylated anti-mouse IgG (Vector laboratories, Inc., CA, USA; 1∶500), rabbit polyclonal anti-Laminin (Sigma-Aldrich, Singapore; 1∶1000) and rabbit polyclonal anti-MyoD (Santa Cruz, USA; 1∶40) for 3 h, the sections were then washed and stained with Streptavidin conjugated Alexa Fluor 488 (Invitrogen; 1∶1000) and goat anti-rabbit Alexa Fluor 594 (Invitrogen; 1∶1000) for 30 min. Nuclei were counterstained with 4′6-diamidino-2-phenylindole (DAPI) (Invitrogen) before mounting with prolong gold anti-fade mounting medium (Invitrogen). Pax7^+^ cells and activated myoblasts (MyoD^+^) that lie underneath the basal lamina, as detected through Laminin staining, were counted and expressed as the percentage of positive nuclei per 100 myofibers. Images were captured using either the Nikon A1Rsi confocal microscope equipped with Photometrics CoolSNAP HQ^2^ camera, or the Leica CTR 6500 microscope equipped with Leica DFC 420 camera and Image Pro Plus software (Media Cybernetics, Bethesda, MD).

### Plasmids

The 3′-UTR of murine *Mstn* (1,448 bp) was PCR amplified using the following primers 5′-AAG CTT GCT TTG CAT TAG GTT-3′, 5′-AAG CTT GCC TTT CAA AAA TG-3′ and cloned as a HindIII fragment into the pMIR-REPORT™ miRNA Expression Luciferase Reporter Vector system (Life Technologies). The construct was sequence verified and named *Mstn* 3′UTR. The predicted miR-27a/b binding site within the *Mstn* 3′ UTR was mutated using a PCR-based mutagenesis approach with combinations of the primers above and the following megaprimer 5′-CCC CTC AAT TTC GAA **GTC ACA GG**T TCA AGC ACC ACA GG-3′, as per the protocol by Picard *et al* 1994 [30]. The mutated *Mstn* 3′ UTR was then cloned as a HindIII fragment into the pMIR-REPORT™ expression reporter vector, sequence verified to confirm mutation of the miR-27a/b binding region and named *Mstn* 3′UTR-mut.

The pcDNA 6.2-GW/± EmGFP expression vector containing mature miR-27a (pcDNA-miR-27a) was used for miR-27a over expression studies. The pcDNA 6.2-GW/± EmGFP empty vector (pcDNA-miR-neg) was used as a control.

The miR-27a promoter (miR-27a pro), miR-27b promoter (miR-27b pro) and the mutant miR-27b promoter reporter construct (miR-27b pro-mut) used in this study were kindly gifted by Dr Xiao Yang (State Key Laboratory of Proteomics, Genetic Laboratory of Development and Diseases, Institute of Biotechnology, Beijing, China) and have been described previously [31], [32].

### Transfections and Luciferase assay

For co-transfection of reporter plasmids and miR-27a over expressing vectors, C2C12 myoblasts were seeded into 24-well plates at a density of 15,000 cells/cm^2^ 24 h before transfection. Proliferating C2C12 myoblasts were co-transfected with 0.1 µg *Mstn* 3′UTR or *Mstn* 3′UTR-mut reporter plasmids and 0.4 µg pcDNA-miR-27a or pcDNA-miR-neg, as a negative control. Transfection was carried out with LF2000 (Invitrogen, USA) according to the manufacturer's protocol. For co-transfection of reporter plasmids and AntagomiRs against miR-27a/b, 50 nM of AntagomiR-27a, AntagomiR-27b or AntagomiR Neg were co-transfected with *Mstn* 3′UTR or *Mstn* 3′UTR-mut using LF2000 (Invitrogen, USA) as per the manufacturer's protocol. After 48 h of transfections, cells were lysed, and luciferase assays were performed on protein extracts using the Dual-Luciferase reporter system (Promega, USA), according to the manufacturer's recommendations. To assess for miR-27a and miR-27b promoter reporter activity, miR-27a pro, miR-27b pro and miR-27b pro-mut constructs were electroporated (GenePulsar MXcell, Bio-rad, Hercules, CA, USA) into 1 million C2C12 cells and grown to confluency. The electroporated C2C12 myoblasts were then replated at a density of 15,000 cells/cm^2^ in 24 well plates and treated with (10 µM) or without (0.05% DMSO) SIS3 in the presence (CMM; 10 ng/ml) or absence (CCM) of Mstn for 24 hrs. Following transfections, cells were lysed, and luciferase assays were performed on protein extracts using the Dual-Luciferase reporter system (Promega, USA), according to the manufacturer's recommendations. Renilla and firefly luciferase signals were detected using the GloMax luminometer (Promega, USA). AntagomiRs (AntagomiR-27a, AntagomiR-27b and AntagomiR Neg) were synthesized by Dharmacon Inc, USA. Synthetic miR-27b mimic and non-targeting miRNA negative control mimic were obtained from Dharmacon. A final concentration of 50 nM each of miR-27b mimic and miRNA negative control were transfected into wild type (WT) and *Smad3*-null mouse primary myoblasts using LF2000 (Invitrogen) as per the manufacturer's protocol. Following transfection primary myoblasts were induced to differentiate under low-serum conditions (DMEM, 2% Horse Serum) for 72 h and then harvested for total RNA isolation and subsequent qPCR analysis.

### RT-PCR and quantitative real-time PCR (qPCR)

Total RNA was extracted using TRIzol reagent according to the manufacturer's protocol (Invitrogen, USA). cDNA was synthesized from 1 µg of total RNA using the iScript cDNA kit (Bio-rad, USA) as per the manufacturer's guidelines. qPCR analysis of precursor-miR-27a/b (Pre-miR-27a/b) and *Mstn* expression was performed using the CFX96 Real-Time System (Bio-rad). Each qPCR reaction (10 µl) contained 3 µl of diluted cDNA, 5 µl of 2 X SsoFast Evagreen (Bio-rad) and primers at a final concentration of 200 nM. All reactions were performed using the following thermal cycle conditions: 98°C for 3 min followed by 45 cycles of a two step reaction, denaturation at 98°C for 3 sec and annealing at 60°C for 20 sec, followed by a denaturation curve from 60°C to 95°C in 5 sec increments of 0.5°C to ensure amplification specificity. To assess for the expression of mature miR-27a and miR-27b, cDNA was synthesized from extracted RNA using the miScript II RT kit (Cat# 218161; Qiagen), as per the manufacturer's instructions. qPCR was then conducted using miR-27a or miR-27b mature miRNA specific miScript forward primers, referred to as Primer Assays (Cat# MS00001351 and Cat# MS00001358 respectively; Qiagen), miScript universal reverse primer (Qiagen) and miScript SYBR Green PCR Kit (Cat# 218075; Qiagen). The expression of mRNAs and miRNAs were normalized to GAPDH and U6, respectively. The following mouse-specific primers were used for qPCR analysis: precursor-miR-27a/b (pre-miR-27a/b) Forward 5′-GCA GGG CTT AGC TGC TTG-3′, Reverse 5′-GGC GGA ACT TAG CCA CTG T-3′; *Mstn* Forward 5′-AGT GGA TCT AAA TGA GGG CAG T-3′, Reverse 5′-GTT TCC AGG CGC AGC TTA C-3′; *U6* Forward 5′-CTC GCT TCG GCA GCA CA-3′ Reverse 5′-AAC GCT TCA CGA ATT TGC GT-3′; *GAPDH* Forward 5′-ACA ACT TTG GCA TTG TGG AA-3′, Reverse 5′-GAT GCA GGG ATG ATG TTC TG-3′.

### Statistical analysis

Statistical analysis was performed using two-tail Student's-t-test and ANOVA. Data are expressed as mean ±SEM and p<0.05 were considered significant. Experimental replicates are described in relevant figure legends.

## Results

### miR-27a/b targets and represses *Mstn* through a miR-27a/b-specific target site in the 3′ UTR of the *Mstn* gene

**Figure 1:**
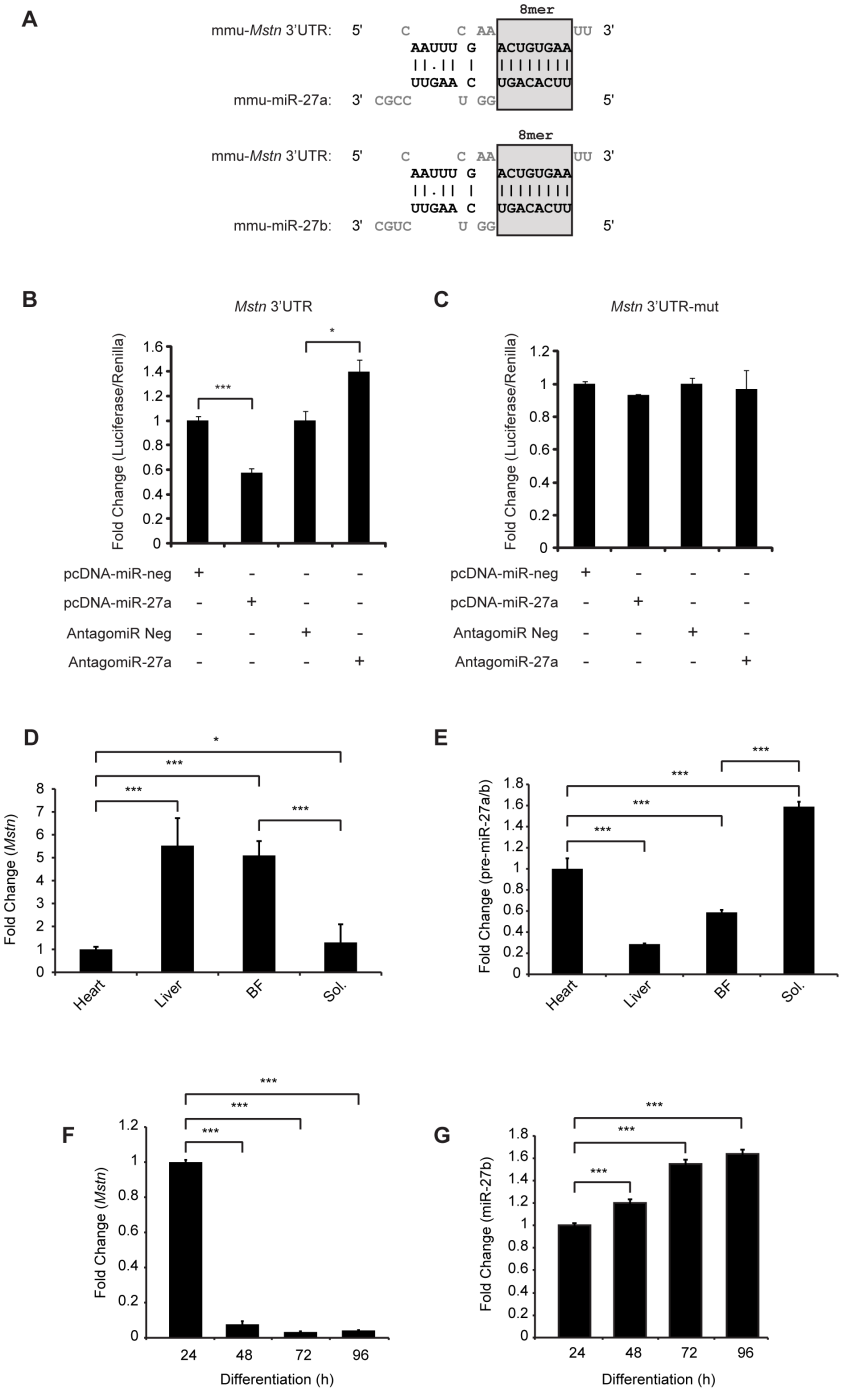
miR-27a/b targets and represses *Mstn* expression. (A) *In silico* analysis, using TargetScan algorithms, showing a 8 mer seed match (grey box) between murine miR-27a (mmu-miR-27a) and miR-27b (mmu-miR-27b) and the miR-27a/b binding site located within the *Mstn* 3′ UTR sequence (mmu-*Mstn* 3′UTR). (B) Assessment of pMIR-REPORT™ luciferase activity in C2C12 myoblasts co-transfected with the *Mstn* 3′UTR reporter construct (*Mstn* 3′UTR) and either control (pcDNA-miR-neg), miR-27a over expression construct (pcDNA-miR-27a), negative control AntagomiR (AntagomiR Neg) or a miR-27a-specific AntagomiR (AntagomiR-27a) for 48h. (C) Assessment of pMIR-REPORT™ luciferase activity in C2C12 myoblasts co-transfected with the mutant *Mstn* 3′UTR reporter construct (*Mstn* 3′UTR-mut), where the miR-27a/b binding site has been mutated, and either control (pcDNA-miR-neg), miR-27a over expression construct (pcDNA-miR-27a), negative control AntagomiR (AntagomiR Neg) or a miR-27a-specific AntagomiR (AntagomiR-27a) for 48h. For all pMIR-REPORT™ transfections, luciferase activity was normalized to Renilla luciferase and expressed as fold change relative to control (pcDNA-miR-neg). Bars represent mean values ± S.E.M (n = 3). *p*<0.05 (*) and *p*<0.001(***). qPCR analysis of *Mstn* mRNA expression (D) and precursor-miR-27a/b (pre-miR-27a/b) expression (E) in Heart, Liver, *M. Biceps femoris* muscle (BF) and *M. Soleus muscle* (Sol.) collected from 4-week-old wild type (WT) mice. Bars represent fold change (relative to Heart) ± S.E.M (n = 3) normalized to *GAPDH* (D) or U6 (E) expression. *p*<0.05 (*) and *p*<0.001(***). qPCR analysis of (F) *Mstn* and (G) miR-27b expression in C2C12 myoblast cultures differentiated across a time course (24 h, 48 h, 72 h and 96 h differentiation). Bars represent fold change (relative to 24 h control) ± S.E.M (n = 3) normalized to either *GAPDH* (F) or U6 (G) expression. *p*<0.001(***).

In agreement with previously published reports [23], [24], analysis with the TargetScan5.1 (http://www.targetscan.org/) algorithm revealed the presence of a single target site for microRNA-27a/b (miR-27a/b) in the 3′UTR of the murine *Mstn* gene (Figure 1A). Importantly, TargetScan analysis revealed an 8 mer seed match, defined as a perfect match to positions 2–8 of the mature miRNA followed by an adenine, between the miR-27a and miR-27b seed sequence and the miR-27a/b binding site in the 3′UTR of the murine *Mstn* gene (Figure 1A). Furthermore the *mstn* 3′UTR miR-27a/b target site was found to be flanked by AU-rich sequences, which act to boost miRNA efficacy [33].

To further study miR-27a/b regulation of *Mstn*, we cloned the 3′ UTR region of the murine *Mstn* gene into the pMIR-REPORT™ miRNA Expression Luciferase Reporter Vector and co-transfected together with a miR-27a over expression vector (pcDNA-miR-27a). A significant reduction in *Mstn* 3′UTR reporter luciferase activity was observed in myoblasts co-transfected with *Mstn* 3′UTR and pcDNA-miR-27a, when compared with myoblasts co-transfected with *Mstn* 3′UTR and control (pcDNA-miR-neg) (Figure 1B). To identify if miR-27a/b target site was responsible for the reduced luciferase activity observed, the putative miR-27a/b binding site in the *Mstn* 3′UTR was mutated. When pcDNA-miR-27a was co-transfected together with the mutant *Mstn* 3′UTR reporter (*Mstn* 3′UTR-mut), no significant reduction in luciferase activity was observed (Figure 1C). Furthermore co-transfection of *Mstn* 3′UTR with a miR-27a-specific AntagomiR (AntagomiR-27a) resulted in significant increase in *Mstn* 3′UTR reporter luciferase activity, over and above that observed in control AntagomiR Neg transfected cells (Figure 1B). The effect of AntagomiR-27a appeared to be specific to the miR-27a/b site in the *Mstn* 3′UTR site, since addition of AntagomiR-27a failed to increase luciferase activity in *Mstn* 3′UTR-mut reporter transfected myoblasts (Figure 1C). These data strongly suggest that miR-27a/b is able to negatively regulate *Mstn* mRNA and that the miR-27a/b target site found within the *Mstn* 3′UTR is critical for miR-27a/b regulation of *Mstn*.

### Correlation between *Mstn* and miR-27a/b expression *in vivo* and *in vitro*

While high levels of Mstn are detected in skeletal muscle, lower levels of Mstn are expressed in white adipose tissue, heart and mammary gland [1]–[3]. Furthermore, Mstn expression is higher in fast-twitch muscles, when compared to slow-twitch muscles [13], [14]. To investigate whether or not miR-27a/b plays a role in regulating tissue-specific *Mstn* expression, we analyzed precursor-miR-27a/b (pre-miR-27a/b) and *Mstn* expression profiles in various tissues. When compared to liver and biceps femoris (BF) muscle, we noted reduced expression of *Mstn* and increased expression of pre-miR-27a/b in the heart (Figure 1D and 1E). On the other hand in tissues where relatively higher levels of *Mstn* were observed, such as liver and BF muscle, lower pre-miR-27a/b expression was detected (Figure 1D and 1E). Consistent with the data published by Allen and Loh [23], we also observed a difference in *Mstn* and pre-miR-27a/b expression between fast-twitch and slow-twitch muscles. qPCR analysis revealed significantly increased *Mstn* mRNA expression, concomitant with reduced pre-miR-27a/b expression in the predominantly fast-twitch BF muscle, when compared to the slow-twitch soleus (Sol) muscle (Figure 1D and 1E).

Next we also compared the expression of *Mstn* and miR-27a/b during differentiation in C2C12 myoblasts. Subsequent qPCR revealed that there was relatively higher expression of *Mstn* at 24 h differentiation, which sharply declined from 48 h differentiation onwards in C2C12 myoblasts (Figure 1F). However in contrast, we observed a gradual increase in miR-27b expression from 24 h through to 96 h differentiation (Figure 1G). Thus, the expression of *Mstn* appears to be inversely associated with miR-27a/b expression during C2C12 myoblast differentiation.

### AntagomiR-mediated blockade of miR-27a/b leads to enhanced Mstn activity

**Figure 2:**
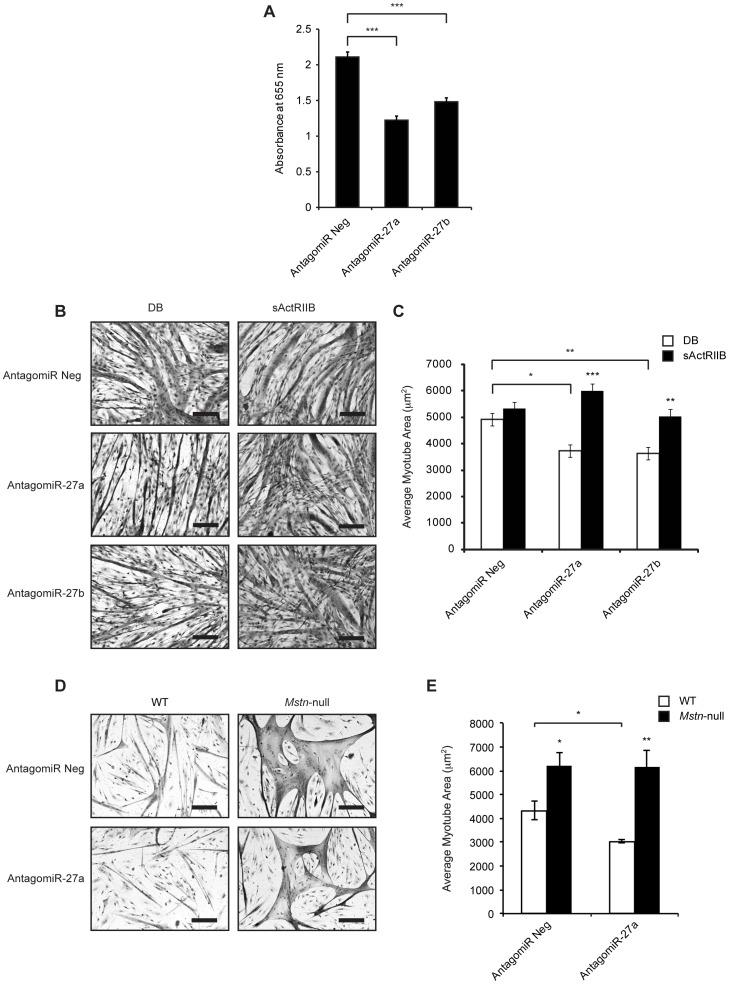
Inhibition of miR-27a and miR-27b results in increased Mstn activity. (A) Analysis of C2C12 myoblast proliferation in cultures treated with conditioned medium collected from C2C12 myoblasts transfected with either the negative control AntagomiR (AntagomiR Neg), miR-27a-specific AntagomiR (AntagomiR-27a) or miR-27b-specific AntagomiR (AntagomiR-27b) for 72 h, as monitored by methylene blue assay. Values represent mean values ± S.E.M (n = 3). *p*<0.001 (***). (B) Representative images of H&E stained AntagomiR Neg, AntagomiR-27a or AntagomiR-27b transfected C2C12 myoblasts after 48 h differentiation, followed by a further 72 h differentiation in the absence (Dialysis buffer; DB) or presence of 3 µg/ml sActRIIB. Scale bars = 100 µm. (C) Quantification of average myotube area (µm^2^) in AntagomiR Neg, AntagomiR-27a or AntagomiR-27b transfected C2C12 myoblasts following 72 h differentiation and treatment without (DB) or with sActRIIB. Average myotube area was calculated from 10 random images per coverslip (n = 3) from three independent experiments. *p*<0.05 (*), *p*<0.01 (**) and *p*<0.001 (***). (D) Representative images of H&E stained AntagomiR Neg or AntagomiR-27a transfected WT and *Mstn*-null primary myoblasts following 48 h differentiation. Scale bars = 100 µm. (E) Quantification of average myotube area (µm^2^) in AntagomiR Neg or AntagomiR-27a transfected WT and *Mstn*-null primary myoblasts following 72 h differentiation. Average myotube area was calculated from 10 random images per coverslip (n = 3). *p*<0.001 (***).

Mstn, as a negative regulator of skeletal muscle growth, has been previously demonstrated to inhibit myoblast proliferation and moreover induce severe myotubular atrophy *in vitro* [5], [8], [9]. Therefore, we next assessed the effect of AntagomiR-27a and AntagomiR-27b transfection on Mstn function during myogenesis. As predicted, transfection of AntagomiR-27a or AntagomiR-27b resulted in reduced expression of miR-27a (Figure S1A) and miR-27b (Figure S1B) respectively, together with increased Mstn expression (Figure S1C). Next we assessed C2C12 myoblast proliferation following treatment with conditioned medium collected from Control (AntagomiR Neg), AntagomiR-27a or AntagomiR-27b transfected C2C12 myoblasts. As shown in Figure 2A, we observed a significant decrease in myoblast proliferation in C2C12 myoblasts treated with conditioned medium collected from AtagomiR-27a and AntagomiR-27b transfected cells, when compared to control (AntagomiR Neg) transfected cells (Figure 2A).

In addition, transfection of either AntagomiR-27a or AntagomiR-27b into differentiating C2C12 myotubes resulted in noticeable myotubular atrophy when compared to AntagomiR Neg transfected myotubes (Figure 2B). Subsequent quantification revealed a significant 24% and 26% decrease in average myotube area in AntagomiR-27a and AntagomiR-27b transfected myotubes respectively, when compared to AntagomiR Neg transfected myotubes (Figure 2C). Consistent with blockade of miR-27a/b and with the development of myotube atrophy a significant increase in *Mstn* expression was observed following transfection of C2C12 myotubes with AntagomiR-27a or AntagomiR-27b (Figure S1D).

To confirm whether or not the myotube atrophy observed following AntagomiR-mediated blockade of miR-27a/b was due to enhanced Mstn function, we next assessed myotube area in AntagomiR-27a and AntagomiR-27b transfected C2C12 myotubes cultures treated together with soluble Activin type IIB receptor (sActRIIB) Mstn antagonist. Treatment of AntagomiR-27a and AntagomiR-27b transfected C2C12 myotubes with sActRIIB rescued the myotubular atrophy observed in the AntagomiR only transfected myotubes (Figure 2B). Subsequent quantification revealed an ∼40% and ∼30% increase in average myotube area, which was similar to that observed in AntagomiR Neg transfected myotubes, in sActRIIB treated AntagomiR-27a and AntagomiR-27b transfected myotubes respectively, when compared to respective vehicle control (Dialysis buffer; DB) treated transfected myotubes (Figure 2C).

In agreement with the results above, AntagomiR-mediated reduction of miR-27a expression (Figure S1E) did not result in any appreciable myotube atrophy in *Mstn*-null mice-derived primary myotube cultures, when compared to AntagomiR Neg transfected primary myotubes (Figure 2D & 2E). However, in contrast, AntagomiR-mediated reduction of miR-27a in primary myotubes cultures isolated from WT mice (Figure S1F) led to elevated Mstn expression (Figure S1G) and observable myotubular atrophy (Figure 2D & 2E), with an ∼32% decrease in average myotube area observed in AntagomiR-27a transfected myotubes, when compared to AntagomiR Neg transfected myotubes (Figure 2E). These results confirm that blockade of miR-27a/b results in myotube atrophy through a mechanism dependent on Mstn.

### miR-27a/b regulates myofiber size and SC function through targeting endogenous *Mstn* expression in skeletal muscle

**Figure 3:**
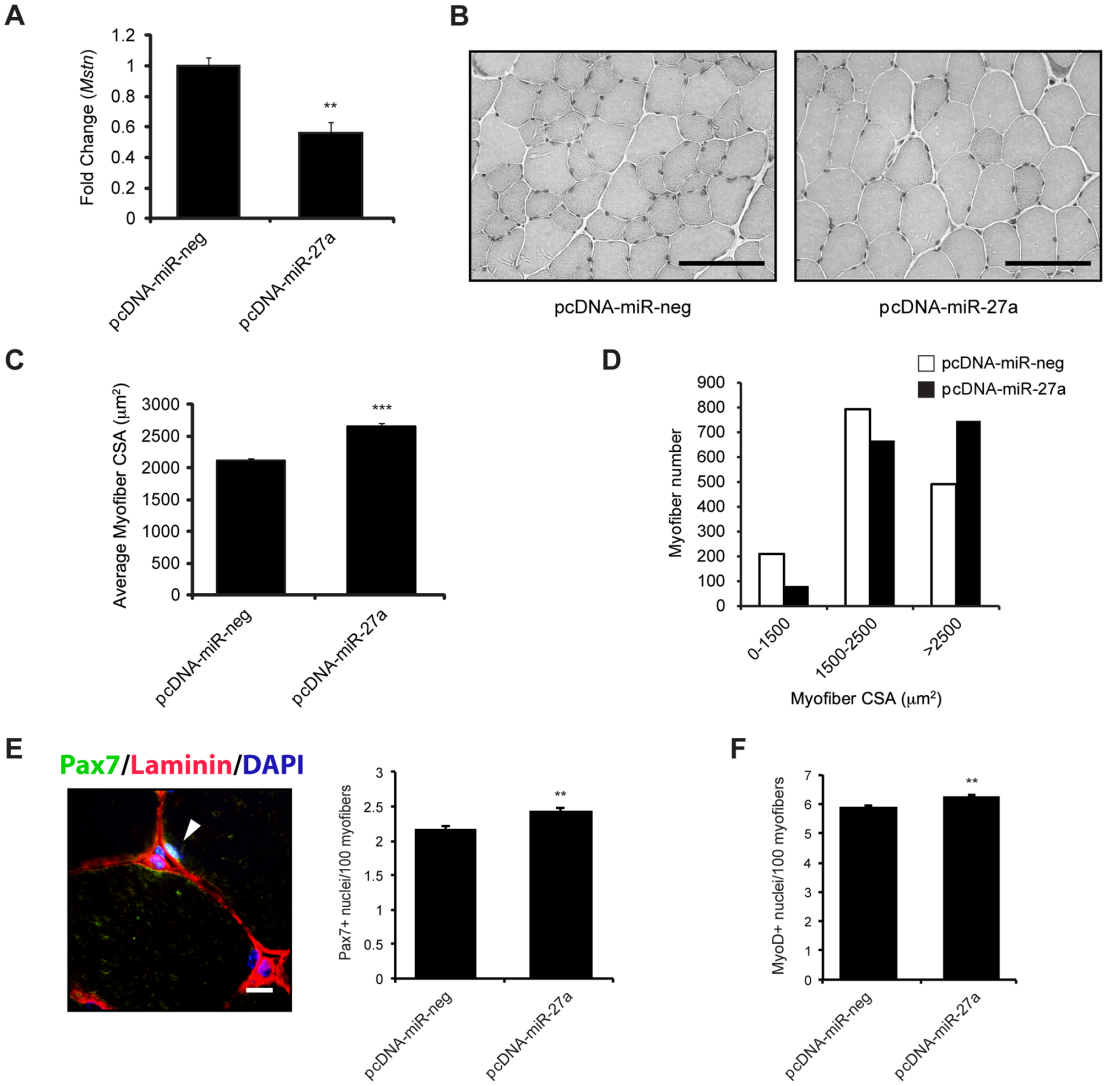
Over expression of miR-27a targets and represses endogenous Mstn expression and function *in vivo*. (A) qPCR analysis of *Mstn* mRNA expression in TA muscle isolated from WT mice (n = 3) 8 days post intramuscular injection and *in vivo* transfection of either control (pcDNA-miR-neg) or miR-27a (pcDNA-miR-27a) over expression constructs. *p*<0.01 (**). (B) Representative images of H&E stained pcDNA-miR-neg and pcDNA-miR-27a *in vivo* transfected TA muscle cross sections from WT mice. Scale bars = 100 µm. (C) Graph showing average myofiber cross sectional area (CSA; µm^2^) in pcDNA-miR-neg and pcDNA-miR-27a *in vivo* transfected TA muscle from WT mice. Average myofiber area was calculated from 10 random images per coverslip (n = 3). *p*<0.001 (***). (D) Frequency distribution of myofiber area (µm^2^) in pcDNA-miR-neg and pcDNA-miR-27a *in vivo* transfected TA muscle from WT mice as calculated from 10 random images per coverslip (n = 3). (E) *Left*: Representative merged immunofluorescence image showing a Pax7^+^ cell (Green; white arrowhead) in a pcDNA-miR-27a *in vivo* transfected TA muscle cross section from WT mice. Sections were also stained for Laminin (Red) and nuclei were counterstained with DAPI (Blue). Scale bar = 10 µm. *Right*: Graph showing the number of Pax7^+^ cells in pcDNA-miR-neg and pcDNA-miR-27a *in vivo* transfected TA muscle from WT mice. Bars represent mean number ± S.E.M of Pax7^+^ cells, per 100 myofibers, from 3 sections each collected from pcDNA-miR-neg and pcDNA-miR-27a transfected WT mice (n = 3). *p*<0.01 (**). (F) Graph showing the number of MyoD^+^ cells in pcDNA-miR-neg and pcDNA-miR-27a *in vivo* transfected TA muscle from WT mice. Bars represent mean number ± S.E.M of MyoD^+^ cells, per 100 myofibers, from 3 sections each collected from pcDNA-miR-neg and pcDNA-miR-27a transfected WT mice (n = 3). *p*<0.01 (**).

To investigate whether miR-27a/b regulates endogenous Mstn levels in skeletal muscle, *M. tibialis anterior* (TA) muscles of WT mice were intramuscularly injected and *in vivo* electroporated with either the pcDNA-miR-27a over expression construct or control (pcDNA-miR-neg). The expression of *Mstn* was quantified by qPCR 8 days post-injection and as shown in Figure 3A overexpression of miR-27a *in viv*o resulted in a significant reduction in *Mstn* expression in TA muscle. Since Mstn is a potent negative regulator of skeletal muscle mass and satellite cell (SC) function [1], [4], [7], [34], we also stained TA muscle serial sections with H&E (Figure 3B) and quantified myofiber cross sectional area (CSA), as well as the percentage of Pax7^+^ and MyoD^+^ cells in TA muscle following miR-27a overexpression. An ∼30% increase in average myofiber CSA was observed in miR-27a overexpressing TA muscle, when compared to the control transfected contralateral TA muscle (Figure 3C). Furthermore we also observed an ∼52% increase in the number of very large myofibers (>2500 µm^2^) and an ∼60% decrease in the number of very small myofibers (<1500 µm^2^) upon *in vivo* overexpression of miR-27a (Figure 3D), which is quite consistent with the increased myofiber CSA and loss of Mstn function. Interestingly, detection of Pax7 and MyoD by immunofluorescence (Figure 3E & S1H) revealed a significant ∼12% and ∼7% increase in the pool of Pax7^+^ cells and activated myoblasts (MyoD^+^) respectively in pcDNA-miR-27a transfected TA muscle, when compared with control-transfected muscle (Figure 3E & 3F).

**Figure 4:**
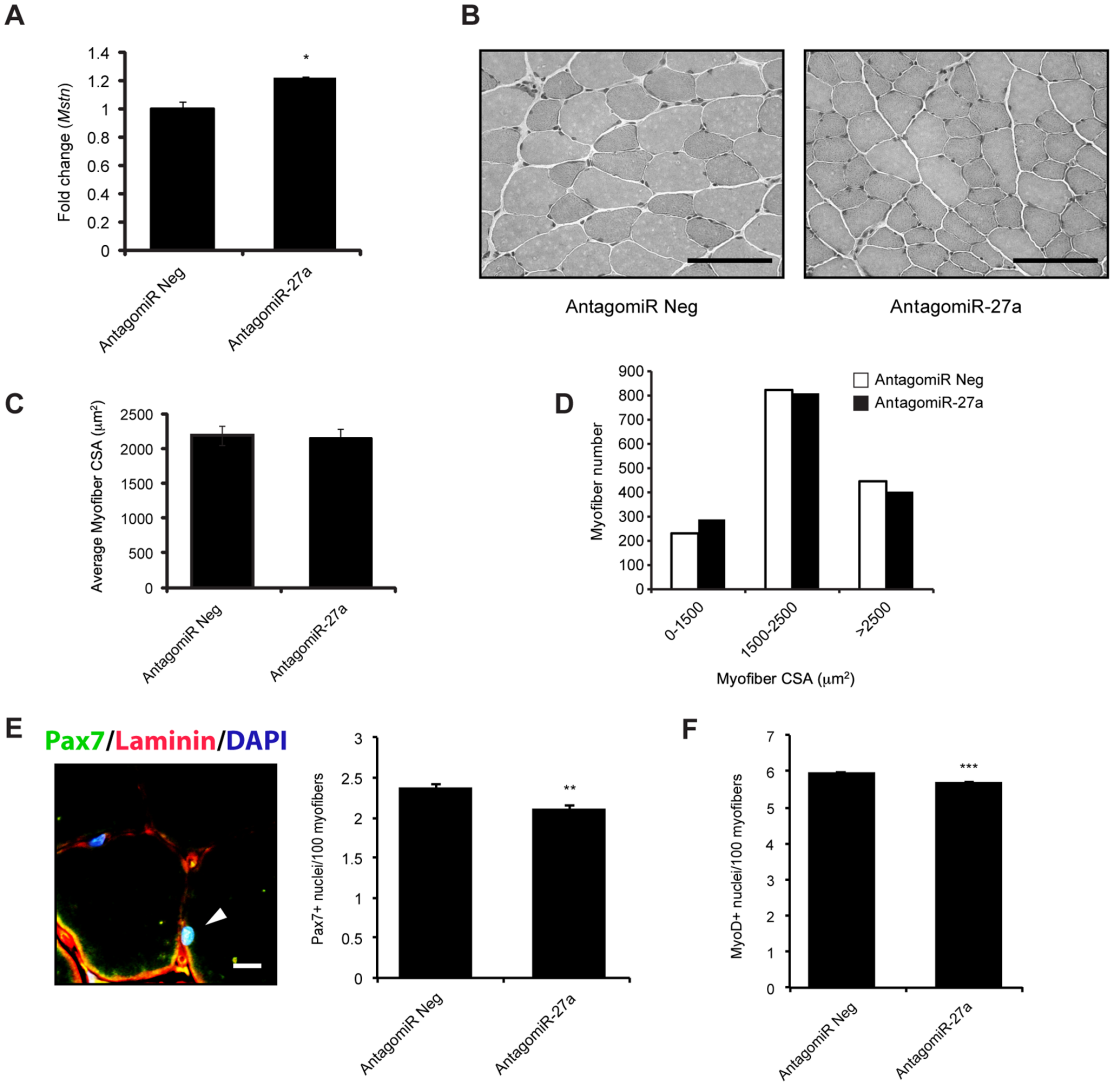
AntagomiR-mediated inhibition of miR-27a enhances endogenous Mstn expression and function *in vivo*. (A) qPCR analysis of *Mstn* mRNA expression in TA muscle isolated from WT mice (n = 3) 8 days post intramuscular injection of either AntagomiR Neg or AntagomiR-27a. *p*<0.05 (*). (B) Representative images of H&E stained AntagomiR Neg and AntagomiR-27a injected TA muscle from WT mice. Scale bars = 100 µm. (C) Graph showing average myofiber CSA (µm^2^) in AntagomiR Neg and AntagomiR-27a injected TA muscle from WT mice. Average myofiber area was calculated from 10 random images per coverslip (n = 3). (D) Frequency distribution of myofiber area (µm^2^) in AntagomiR Neg and AntagomiR-27a injected TA muscle from WT mice as calculated from 10 random images per coverslip (n = 3). (E) *Left*: Representative merged immunofluorescence image showing a Pax7^+^ cell (Green; white arrowhead) in an AntagomiR Neg injected TA muscle cross section from WT mice. Sections were also stained for Laminin (Red) and nuclei were counterstained with DAPI (Blue). Scale bar = 10 µm. *Right*: Graph showing the number of Pax7^+^ cells in AntagomiR Neg and AntagomiR-27a injected TA muscle from WT mice. Bars represent mean number ± S.E.M of Pax7^+^ cells, per 100 myofibers, from 3 sections each collected from AntagomiR Neg and AntagomiR-27a injected WT mice (n = 3). *p*<0.01 (**). (F) Graph showing the number of MyoD^+^ cells in AntagomiR Neg and AntagomiR-27a injected TA muscle from WT mice. Bars represent mean number ± S.E.M of MyoD^+^ cells, per 100 myofibers, from 3 sections each collected from AntagomiR Neg and AntagomiR-27a injected WT mice (n = 3). *p*<0.001 (***).

To further confirm regulation of endogenous *Mstn* expression by miR-27a, we also injected either an AntagomiR specific for miR-27a (AntagomiR-27a) or a non-silencing negative control AntagomiR (AntagomiR Neg) into TA muscle of WT mice. The expression of *Mstn* was assessed 8 days post-injection and consistent with reduced miR-27a, *Mstn* expression was significantly up regulated, albeit modestly, upon AntagomiR-mediated blockade of miR-27a *in vivo*, when compared to AntagomiR Neg transfected contralateral TA muscle. (Figure 4A). We further stained TA muscle serial sections with H&E (Figure 4B) and although no significant difference was noted in average myofiber CSA (Figure 4C), we did observe an ∼25% increase in the number of very small myofibers (<1500 µm^2^) and an ∼10% decrease in the number of very large myofibers (>2500 µm^2^) (Figure 4D), which is quite consistent with the elevated *Mstn* expression detected. Furthermore, detection of Pax7 and MyoD by immunofluorescence (Figure 4E & S1H) revealed a significant ∼11% and ∼4% decrease in the pool of Pax7^+^ cells and activated myoblasts (MyoD^+^) respectively in AntagomiR-27a transfected TA muscle, when compared with AntagomiR Neg-transfected muscle (Figure 4E & 4F).

### Increased *Mstn* expression observed in the absence of Smad3 is due to reduced miR-27a/b expression

**Figure 5:**
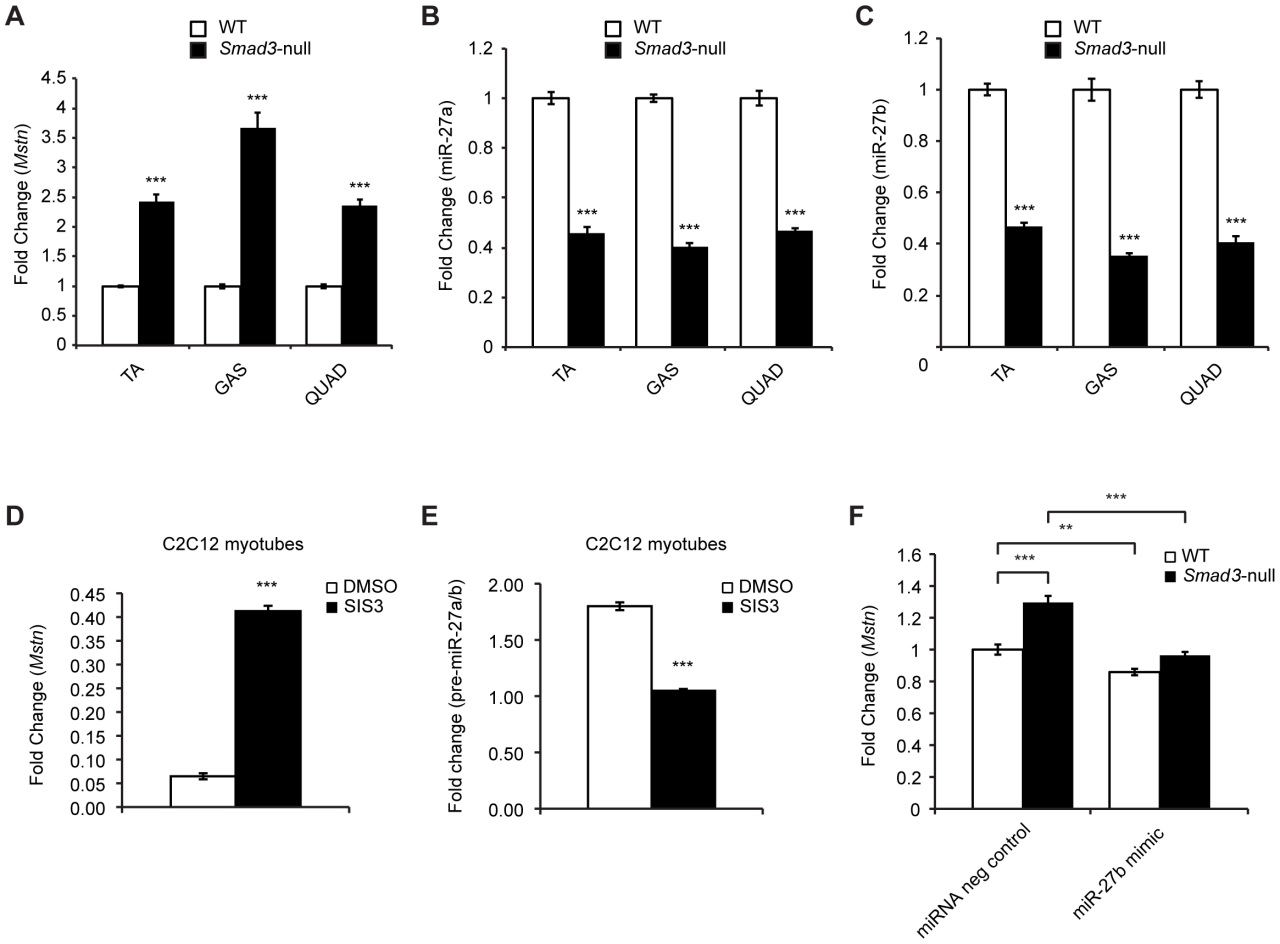
Increased *Mstn* expression in *Smad3*-null mice is due to reduced miR-27a/b expression. qPCR analysis of (A) *Mstn*, (B) miR-27a and (C) miR-27b expression in *M. Tibialis anterior* muscle (TA), *M. Gastrocnemius* muscle (GAS) and *M. Quadriceps muscle* (QUAD) isolated from WT and *Smad3*-null mice. Bars represent fold change (relative to respective WT control) ± S.E.M (n = 3) normalized to either *GAPDH* (A) or U6 (B & C) expression. *p*<0.001 (***). (D) qPCR analysis of *Mstn* expression in 48 h differentiated C2C12 myotubes treated without (0.05% DMSO) or with SIS3 (10 µM) for 24 h. *p*<0.001 (***). (E) qPCR analysis of pre-miR-27a/b expression in 48 h differentiated C2C12 myotubes treated without (0.05% DMSO) or with SIS3 (10 µM) for 24 h. *p*<0.001 (***). (F) qPCR analysis of *Mstn* in 72 h differentiated primary myoblast cultures isolated from WT and *Smad3*-null mice that were transfected with either non targeting miRNA negative control (miRNA neg control) or miR-27b-specific mimic (miR-27b mimic). Bars represent fold change (relative to WT miRNA Neg control transfected myoblasts) ± S.E.M (n = 3) normalized to *GAPDH* expression. *p*<0.01 (**) and *p*<0.001 (***).

Smad3-null mice display severe muscle atrophy, which has been attributed to elevated endogenous levels of Mstn detected in *Smad3*-null mice [35]. Therefore we next wanted to test whether or not the increased Mstn levels observed in *Smad3*-null mice was due to reduced miR-27a/b expression. Consistent with previously published data, qPCR analysis revealed a significant increase in *Mstn* expression in TA, GAS and QUAD muscles isolated from *Smad3*-null mice, when compared to WT controls (Figure 5A). Importantly, the elevated *Mstn* expression was associated with a significant decrease in both mature miR-27a and miR-27b expression in all muscle tissues isolated from *Smad3*-null mice, as compared to WT mice (Figure 5B & 5C). Similarly, C2C12 myotubes treated with Specific Inhibitor of Smad3 (SIS3), a compound previously shown to specifically inhibit Smad3 function via suppressing Smad3 phosphorylation [36], displayed significantly increased *Mstn* expression concomitant with reduced pre-miR-27a/b (Figure 5D & 5E). To confirm that the reduced expression of miR-27 was responsible for the elevated Mstn expression detected in *Smad3*-null mice we next assessed Mstn expression between WT and *Smad3*-null mice primary myoblast cultures following transfection of a miR-27b-specific mimic. As expected *Mstn* expression was significantly elevated in *Smad3*-null cultures, when compared to WT cultures (Figure 5F). Importantly, transfection of the miR-27b mimic reduced the expression of *Mstn* back to levels comparable to that observed in WT controls (Figure 5F), suggesting that reduced miR-27a/b expression may be responsible for the increased levels of Mstn observed in *Smad3*-null mice.

### Mstn upregulates miR-27a/b expression through a Smad3-dependent mechanism to negatively auto-regulate its own expression

**Figure 6:**
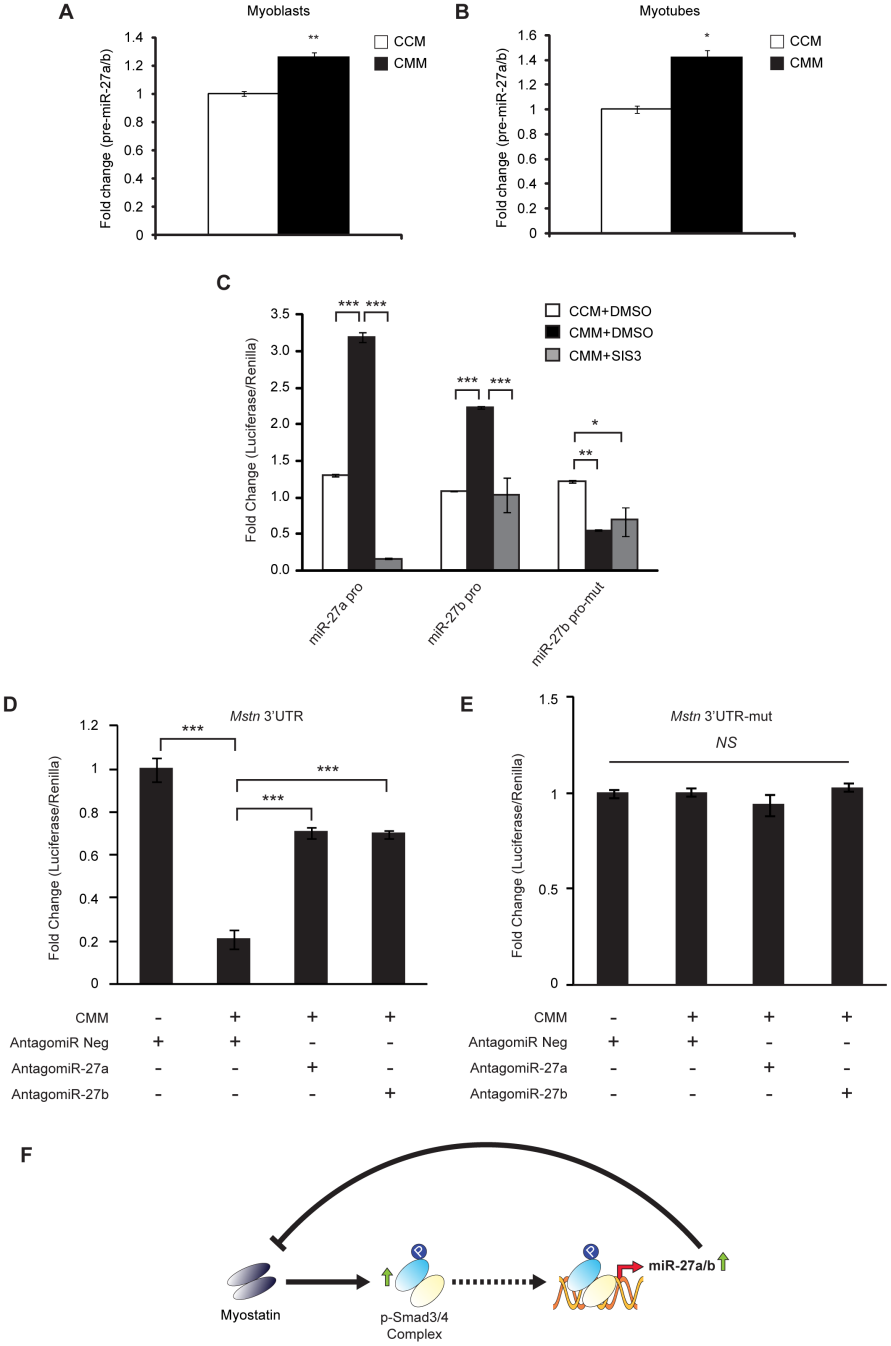
Mstn treatment up regulates miR-27a/b expression via Smad3 to negatively auto-regulate it's own expression. qPCR analysis of pre-miR-27a/b expression in C2C12 myoblasts (A) and 48 h differentiated C2C12 myotubes (B) following 12 h treatment with conditioned medium from either control CHO cells (CCM) or from CHO-cells designed to produce and secrete Mstn protein (CMM). Bars represent fold change (relative to respective CCM control) ± S.E.M (n = 3) normalized to U6 expression. *p*<0.05 (*) and *p*<0.01 (**). (C) Assessment of miR-27a and miR-27b promoter-reporter luciferase activity in C2C12 myoblasts transfected with the miR-27a promoter (miR-27a pro), miR-27b promoter (miR-27b pro) or a mutant miR-27b promoter reporter construct, where the smad binding site has been mutated (miR-27b pro-mut). Transfected C2C12 myoblasts were treated without (CCM) or with CMM in the absence (0.05% DMSO) or presence of SIS3 (10 µM) for 24 h prior to assessment of luciferase activity. All luciferase activity was normalized to Renilla luciferase and expressed as fold change relative to respective controls (CCM+DMSO). Bars represent mean values ± S.E.M (n = 3). *p*<0.05 (*), *p*<0.01 (**) and *p*<0.001 (***). (D) Assessment of pMIR-REPORT™ luciferase activity in C2C12 myoblasts co-transfected with *Mstn* 3′UTR and either AntagomiR Neg, AntagomiR-27a or AntagomiR-27b in the absence (−) or presence (+) of CMM. Bars represent mean values ± S.E.M (n = 3). *p*<0.001 (***). (E) Assessment of pMIR-REPORT™ luciferase activity in C2C12 myoblasts co-transfected with *Mstn* 3′UTR-mut and either AntagomiR Neg, AntagomiR-27a or AntagomiR-27b in the absence (−) or presence (+) of Mstn protein (CMM). Bars represent mean values ± S.E.M (n = 3). All luciferase activity was normalized to Renilla luciferase and expressed as fold change relative to control (CMM - and AntagomiR Neg +). (F) Based on the data presented in this current manuscript we propose that upon Mstn-mediated receptor activation Smad3 up-regulates the expression of miR-27a/b, which in turn leads to reduced *Mstn* expression and impaired Mstn function, thus forming the basis of a novel negative Mstn auto-regulatory loop in muscle.

The data presented above suggested to us that Smad3 may play an important role in regulating basal miR-27a/b expression in muscle. Since Mstn is known to activate Smad3, we further hypothesized that Mstn may signal to up regulate the expression of miR-27a/b in muscle. To determine whether Mstn regulates miR-27a/b expression, C2C12 myoblasts and 48 h differentiated myotubes were treated with conditioned medium containing eukaryotic produced CHO-cell secreted Mstn protein (CMM) for 12 h. Pre-miR-27a/b expression was quantified by qPCR and as can be seen in Figure 6A & 6B, the expression of pre-miR-27a/b was significantly increased in both C2C12 myoblasts and myotubes upon treatment with CMM, when compared with cells treated with conditioned medium collected from control CHO cells (CCM) (Figure 6A & 6B). These data confirm that Mstn is able to positively regulate miR-27a/b expression in muscle.

To confirm whether Smad3 is involved in Mstn regulation of miR-27a/b, C2C12 myoblasts were transfected with either the miR-27a promoter (miR-27a pro), miR-27b promoter (miR-27b pro) or a mutant miR-27b promoter reporter construct, where the smad binding site has been mutated (miR-27b pro-mut) and subjected to treatment with CMM. Treatment with CMM resulted in a significant increase in promoter-reporter luciferase activity in myoblasts transfected with either the miR-27a or miR-27b promoter constructs (Figure 6C); however, no significant increase in luciferase activity was observed in C2C12 myoblasts transfected with the mutated miR-27b promoter construct following CMM treatment (Figure 6C). Transfected myoblasts were also subjected to treatment with both CMM and SIS3. As shown in Figure 6C, addition of SIS3 was able to partially rescue the increased miR-27a- and miR-27b-promoter-reporter luciferase activity observed following treatment with CMM alone (Figure 6C). Therefore these data confirm that Smad3 plays a critical role in the ability of Mstn to up regulate miR-27a/b expression.

Next we assessed whether or not the increased miR-27a/b, due to CMM treatment, would in turn target and repress *Mstn* expression. To test this C2C12 myoblasts transfected with the *Mstn* 3′UTR reporter were subjected to treatment with CMM. As shown in Figure 6D, treatment of *Mstn* 3′UTR reporter transfected myoblasts with CMM resulted in an ∼50% reduction in *Mstn* 3′UTR reporter activity. The ability of CMM to reduce *Mstn* 3′UTR reporter activity appeared to be dependent on miR-27a/b function, as AntagomiR-mediated inhibition of miR-27a/b, as well as mutation of the miR-27a/b binding site in the *Mstn* 3′UTR, prevented CMM-mediated inhibition of *Mstn* expression (Figure 6D & 6E). Taken together these data highlight a novel negative auto-regulatory mechanism through which Mstn signals to regulate it's own expression (Figure 6F).

## Discussion

In the present study we have further characterized the role of miR-27a/b in regulating Mstn expression and activity. Evidence presented here confirms that *Mstn* is indeed a target of miR-27a/b both *in vitro* and *in vivo*. Consistent with previous reports [23], [24], we show that over expression of miR-27a results in reduced *Mstn* 3′UTR reporter activity, which is blocked upon mutation of the miR-27a/b binding site in the *Mstn* 3′ UTR. Furthermore, over expression of miR-27a *in vivo* led to decreased *Mstn* expression concomitant with myofiber hypertrophy and increased numbers of Pax7^+^ cells and activated myoblasts (MyoD^+^); quite consistent with the fact that loss of *Mstn* leads to increased muscle mass and enhanced satellite cell number, activation and self-renewal [1], [4], [34]. Previously published work has revealed that hypertrophy of skeletal muscle may occur independent of satellite cell function [37]. Consistent with this, hypertrophy of skeletal muscle induced upon blockade of Mstn, was also shown to occur in the absence of satellite cells [38], [39]. Nevertheless, as satellite cells play a critical role during skeletal muscle regeneration [37] and that loss of *Mstn* leads to enhanced satellite cell activation, self-renewal and accelerated skeletal muscle regeneration [7], [34] we strongly believe that the increased numbers of Pax7^+^ and MyoD^+^ cells observed following over expression of miR-27a is due to loss of Mstn. Recent work from Crist *et al* has shown that miR-27 is able to down regulate Pax3 protein levels, without affecting the levels of Pax7 [40]. However, we now show that over expression of miR-27 *in vivo* leads to increased numbers of Pax7^+^ cells. It is important to highlight that previously published work from our lab revealed that Mstn is a potent negative regulator of Pax7 expression during myogenesis. [26]. Therefore, the increase in Pax7^+^ cells observed in response to over expression of miR-27 is most likely due to miR-27-mediated inhibition of *Mstn* as opposed to direct regulation of Pax7 by miR-27. In addition to over expression studies, we now show that blockade of miR-27a, through addition of an AntagomiR specific for miR-27a, results in enhanced *Mstn* 3′UTR reporter activity. AntagomiR-mediated blockade of miR-27a and miR-27b not only up regulated *Mstn* expression but also significantly reduced C2C12 myoblast proliferation. These data are consistent with previously published reports demonstrating that excess Mstn inhibits myoblast proliferation [5] and with a recent report, which shows that addition of miR-27a mimics results in decreased *Mstn* mRNA concomitant with an increase in the number of proliferating C2C12 myoblasts [24]. In addition to controlling myoblast growth excess Mstn has been shown to promote skeletal muscle wasting *in vitro* and *in vivo* [10], [11]. In agreement with this, we observed significantly increased expression of *Mstn* together with pronounced myotubular atrophy upon AntagomiR-mediated inhibition of miR-27a and miR-27b. This effect appeared to be dependent on increased *Mstn* as sActRIIB-mediated blockade of Mstn rescued the atrophy phenotype and moreover primary myotube cultures isolated from *Mstn*-null mice were resistant to AntagomiR-induced myotube atrophy. It is important to mention that we noted differences in the effects of AntagomiR-27a and AntagomiR-27b on *Mstn* expression between proliferating C2C12 myoblasts and differentiated C2C12 myotube cultures (compare Figure S1C to Figure S1D). Although we do observe a significant increase in *Mstn* expression in both C2C12 myoblasts and myotubes transfected with either AntagomiR-27a or AntagomiR-27b the increase in *Mstn* was more significant in AntagomiR transfected myotube cultures, when compared to proliferating myoblasts. At this stage we do not know why there are differences in AntagomiR-27a- and AntagomiR-27b-mediated regulation of *Mstn* between myoblasts and myotubes, however we speculate that the AntagomiR effect might be more persistent in myotube cultures when compared to proliferating myoblasts. Blockade of miR-27a *in vivo* also resulted in significantly increased Mstn expression, which was associated with decreased myofiber CSA. Unlike the dramatic phenotype observed *in vitro*, AntagomiR-mediated blockade of miR-27a only resulted in minor muscle atrophy. However, it is important to mention that we only observed a slight increase in Mstn expression *in vivo* upon AntagomiR injection, which we suggest may account for the subtle atrophy phenotype observed. Nevertheless, we did note a significant reduction in the numbers of Pax7^+^ cells and activated myoblasts (MyoD^+^) upon AntagomiR-mediated blockade of miR-27a *in vivo*, further confirming that miR-27 is able to regulate *Mstn*. Taken together these data presented here strongly support that miR-27a/b negatively regulates both *Mstn* expression and function *in vitro* and *in vivo*. Importantly, in our current experiments we did not observe any significant difference in the ability of miR-27a or miR-27b to regulate *Mstn* expression or activity. Given that miR-27a and miR-27b have the same “seed” sequence, UGACACU, which recognizes complementary sequences in the 3′UTRs of target genes, it is not surprising that we found no difference in the ability of miR-27a or miR-27b to regulate *Mstn*.

To date several studies have shown that Mstn expression is relatively higher in fast twitch muscle fibers, when compared to slow twitch muscle fibers [13], [14]. Recently published evidence suggests that miR-27 may play a role in regulating skeletal muscle fiber type-specific expression of Mstn [23]. Specifically, work from Allen and Loh revealed that miR-27a and miR-27b fast-twitch and slow-twitch muscle-specific expression was complementary to that of Mstn. In the current manuscript we also found a similar trend in miR-27a/b and Mstn expression between fast and slow muscle fiber types. Interestingly, here we further show that miR-27a/b and Mstn expression was inversely associated between different tissues. Specifically, when compared to heart tissue, we noted higher Mstn expression, concomitant with reduced expression of miR-27a/b in liver tissue. It is noteworthy to mention that although expression of *Mstn* has been detected in Liver previously [41], [42], high expression of *Mstn* in the Liver, as shown here, has not been previously reported. We speculate that variations in the expression of *Mstn* detected in Liver tissue between various studies may be due to differences in the sensitivity of the techniques used to assess for *Mstn* expression. In addition to regulating fiber type- and tissue-specific *Mstn* expression, we also show that miR-27a/b could potentially regulate *Mstn* mRNA levels during myogenic differentiation *in vitro*. As differentiation ensued we noted a decrease in Mstn expression, consistent with previous reports [43], [44], concomitant with a steady increase in miR-27 expression. These data are in agreement with a recently published report by Chen *et al*, which shows a similar increase in miR-27 expression and associated decrease in *Mstn* expression during myogenic differentiation [45]. As Mstn is a potent negative regulator of myoblast differentiation [6], we speculate that the elevated miR-27 expression may function to inhibit *Mstn* expression thus allowing for myogenic differentiation to proceed. Although it is tempting to suggest that epigenetic mechanisms (such as miR-27) could be responsible for regulating *Mstn* expression during differentiation, we noted that the increase in miR-27 expression during differentiation might not be enough to account for the dramatic drop in *Mstn* expression observed. These data suggest that additional factors may play a role in inhibiting *Mstn* during differentiation. One likely candidate, that may be responsible for regulating Mstn expression during differentiation, is Smad7. Consistent with this, previously published work from our lab clearly demonstrates that Smad7 is able to inhibit *Mstn* expression [16]. Moreover, Kollias *et al* have previously demonstrated that the expression of Smad7 is elevated during differentiation and that over expression of Smad7 results in enhanced myogenic differentiation and rescue of Mstn-mediated inhibition of differentiation [46]. However, further work will need to be performed to confirm this. A role for miR-27 in regulating myogenic differentiation is not novel, in fact Crist *et al* recently demonstrated that miR-27b is able to negatively regulate Pax3 protein levels in adult muscle satellite cells to allow for timely entry into the myogenic differentiation program [40]. Therefore, taken together these data suggest that posttranscriptional regulation of *Mstn* mRNA by miR-27 plays a critical role in controlling timely tissue-specific expression/activity of Mstn during development.

Recently, we have shown that *Smad3*-null mice have elevated expression of Mstn and not surprisingly pronounced skeletal muscle atrophy [35]. Here we have investigated if miR-27a/b could be responsible for the increased Mstn expression observed in *Smad3*-null mice; and in agreement with increased Mstn expression we find reduced miR-27a and miR-27b expression in *Smad3*-null mice. More importantly, mimic-mediated over expression of miR-27b in *Smad3*-null mice was able to reduce *Mstn* expression back to levels comparable to WT mice. Therefore we speculate that loss of *Smad3* leads to reduced miR-27a/b expression, which in turn increases *Mstn* mRNA stability and or translation leading to enhanced skeletal muscle wasting. These data, together with the fact that specific inhibitor of Smad3 (SIS3) treatment was able to significantly reduce miR-27a/b expression, suggest that Smad3 plays an important role in regulating endogenous miR-27a/b expression. Further support for Smad3 regulation of miR-27 is seen in published work from Sun *et al*, which revealed the presence of a Smad binding element in the miR-24-2/miR-23a/miR-27a cluster upstream regulatory sequence and that the Smad binding site was critical for TGF-β1-mediated inhibition of miR-24-2/miR-23a/miR-27a [31].

Interestingly, we now show for the first time that Mstn is able to up regulate the expression of miR-27a/b. Furthermore, Smad3 is critical for Mstn regulation of miR-27a/b as either mutation of the Smad binding site or treatment with SIS3 ablated the Mstn-mediated response. Mstn has been previously shown to negatively feedback to block its own expression/activity, through mechanisms involving inhibition of Mstn proteolytic processing and Smad7-dependent inhibition of Mstn expression [12], [16]. Here we now describe an independent mechanism through which Mstn regulates it's own expression. Specifically, addition of exogenous Mstn resulted in increased miR-27a/b expression, which in turn led to reduced *Mstn* 3′ UTR activity. This mechanism was dependent on miR-27a/b, as either blockade of miR-27a/b or mutation of the miR-27a/b binding site in the *Mstn* 3′ UTR prevented Mstn feedback regulation. These data, together with previously published work, suggest that there are independent auto-regulatory mechanisms through which Mstn regulates it's own activity; given the fact that Mstn is a potent negative regulator of skeletal muscle myogeneis, we speculate that such mechanisms are in place to allow for timely regulation of myogenesis.

In summary, we provide further evidence to support a role for miR-27 in regulating Mstn expression. Evidence suggests that miR-27a and miR-27b play an important role in controlling tissue-specific and muscle fiber type-specific expression of Mstn and regulating Mstn function during myogenesis. Furthermore, we now show that miR-27a/b forms the basis of a novel negative auto-regulatory mechanism through which Mstn inhibits it's own expression in muscle.

## Supporting Information

Figure S1 **AntagomiR-mediated inhibition of miR-27a/b and enhanced expression of** ***Mstn*** **.** (A) qPCR analysis of miR-27a expression in C2C12 myoblasts following transfection of AntagomiR Neg or AntagomiR-27a. Bars represent fold change (relative to AntagomiR Neg control) ± S.E.M (n = 3) normalized to U6 expression. *p*<0.001 (***). (B) qPCR analysis of miR-27b expression in C2C12 myoblasts following transfection of AntagomiR Neg or AntagomiR-27b. Bars represent fold change (relative to AntagomiR Neg control) ± S.E.M (n = 3) normalized to U6 expression. *p*<0.001 (***). (C) qPCR analysis of *Mstn* expression in C2C12 myoblasts following transfection of AntagomiR Neg, AntagomiR-27a or AntagomiR-27b. Bars represent fold change (relative to AntagomiR Neg control) ± S.E.M (n = 3) normalized to *GAPDH* expression. *p*<0.05 (*) and *p*<0.01 (**). (D) qPCR analysis of *Mstn* expression in differentiated C2C12 myotubes following transfection of AntagomiR Neg, AntagomiR-27a or AntagomiR-27b. Bars represent fold change (relative to AntagomiR Neg control) ± S.E.M (n = 3) normalized to *GAPDH* expression. *p*<0.001 (***). (E) qPCR analysis of miR-27a expression in differentiated primary myoblast cultures from *Mstn*-null mice following transfection of AntagomiR Neg or AntagomiR-27a. Bars represent fold change (relative to AntagomiR Neg control) ± S.E.M (n = 3) normalized to U6 expression. *p*<0.001 (***). qPCR analysis of miR-27a (F) and *Mstn* (G) expression in differentiated primary myoblast cultures from WT mice following transfection of AntagomiR Neg or AntagomiR-27a. Bars represent fold change (relative to AntagomiR Neg control) ± S.E.M (n = 3) normalized to U6 (F) or *GAPDH* (G) expression. *p*<0.001 (***). (H) *Upper panel*: Representative immunofluorescence images showing Pax7^+^ cells (Green; white arrowheads) in an *in vivo* transfected TA muscle cross section from WT mice. Nuclei were counterstained with DAPI (Blue) and a Pax7/DAPI merged image is also shown. Scale bars = 10 µm. *Lower panel*: Representative immunofluorescence images showing MyoD^+^ cells (Red; white arrowheads) in an *in vivo* transfected TA muscle cross section from WT mice. Nuclei were counterstained with DAPI (Blue) and a MyoD/DAPI merged image is also shown. Scale bars = 100 µm.
(TIF)
