# Supplementary figures and images for: Negative Auto-Regulation of Myostatin Expression is Mediated by Smad3 and MicroRNA-27
Source: PLoS One. 2014 Jan 31;9(1):e87687. doi: 10.1371/journal.pone.0087687 (PMC3909192; doi:10.1371/journal.pone.0087687)

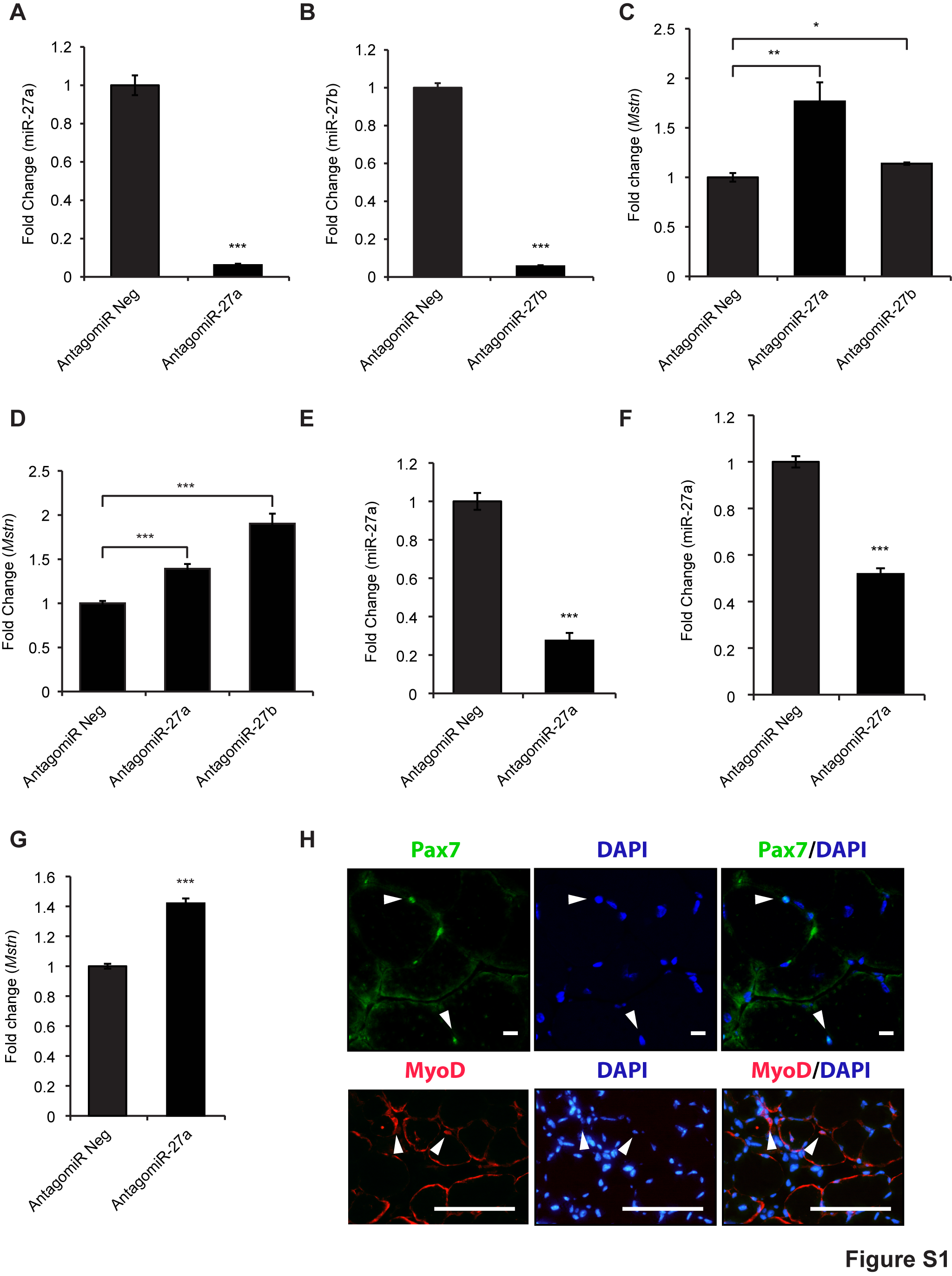

Supplement: Figure S1 — AntagomiR-mediated inhibition of miR-27a/b and enhanced expression of Mstn . (A) qPCR analysis of miR-27a expression in C2C12 myoblasts following transfection of AntagomiR Neg or AntagomiR-27a. Bars represent fold change (relative to AntagomiR Neg control) ± S.E.M (n = 3) normalized to U6 expression. p<0.001 (***). (B) qPCR analysis of miR-27b expression in C2C12 myoblasts following transfection of AntagomiR Neg or AntagomiR-27b. Bars represent fold change (relative to AntagomiR Neg control) ± S.E.M (n = 3) normalized to U6 expression. p<0.001 (***). (C) qPCR analysis of Mstn expression in C2C12 myoblasts following transfection of AntagomiR Neg, AntagomiR-27a or AntagomiR-27b. Bars represent fold change (relative to AntagomiR Neg control) ± S.E.M (n = 3) normalized to GAPDH expression. p<0.05 (*) and p<0.01 (**). (D) qPCR analysis of Mstn expression in differentiated C2C12 myotubes following transfection of AntagomiR Neg, AntagomiR-27a or AntagomiR-27b. Bars represent fold change (relative to AntagomiR Neg control) ± S.E.M (n = 3) normalized to GAPDH expression. p<0.001 (***). (E) qPCR analysis of miR-27a expression in differentiated primary myoblast cultures from Mstn-null mice following transfection of AntagomiR Neg or AntagomiR-27a. Bars represent fold change (relative to AntagomiR Neg control) ± S.E.M (n = 3) normalized to U6 expression. p<0.001 (***). qPCR analysis of miR-27a (F) and Mstn (G) expression in differentiated primary myoblast cultures from WT mice following transfection of AntagomiR Neg or AntagomiR-27a. Bars represent fold change (relative to AntagomiR Neg control) ± S.E.M (n = 3) normalized to U6 (F) or GAPDH (G) expression. p<0.001 (***). (H) Upper panel: Representative immunofluorescence images showing Pax7+ cells (Green; white arrowheads) in an in vivo transfected TA muscle cross section from WT mice. Nuclei were counterstained with DAPI (Blue) and a Pax7/DAPI merged image is also shown. Scale bars = 10 µm. Lower panel: Representative immunoflu [file pone.0087687.s001.tif]
